# Postnatal Care Service Utilization and Associated Factors Among Women in Rural Afghanistan: A Cross‐Sectional Analysis of the 2022–2023 Multiple Indicator Cluster Survey

**DOI:** 10.1002/hsr2.72913

**Published:** 2026-07-27

**Authors:** Essa Tawfiq, Muhammad Haroon Stanikzai, Zainab Ezadi, Fateme Dadras, Hadia Sayam, Omid Dadras

**Affiliations:** ^1^ The Kirby Institute, UNSW Sydney Sydney Australia; ^2^ Department of Public Health, Faculty of Medicine Kandahar University Afghanistan; ^3^ Master of Science in Midwifery Reproductive Health Kabul Afghanistan; ^4^ Department of Obstetrics and Gynecology, Faculty of Medicine University of Medical Sciences Karaj Iran; ^5^ Department of Para‐clinic, Faculty of Medicine Malalay University Afghanistan; ^6^ Research Centre for Child Psychiatry University of Turku Turku Finland

**Keywords:** Afghanistan, determinants, postnatal care, rural regions, women

## Abstract

**Background and Aims:**

Although Afghanistan has achieved notable reductions in maternal and overall mortality over the past two decades, significant disparities remain between urban and rural populations. This study assessed the magnitude and determinants of postnatal care (PNC) service utilization among women in rural Afghanistan.

**Methods:**

This study used secondary data from a nationwide representative sample of the 2022–2023 Afghanistan Multiple Indicator Cluster Survey (MICS). The study included ever‐married women aged 15–49 years living in rural areas who had at least one live birth in the 2 years preceding the survey. The outcome variable was defined as a binary variable on PNC service utilization (≥ 1 PNC visit equals 1, and zero otherwise). Bivariate and multivariable logistic regression models were fitted to identify factors associated with PNC service utilization.

**Results:**

Of 10,644 women, only 14.4% (95% CI 13.3–15.4%) of women had at least one PNC visit. PNC service utilization was significantly more likely among women who delivered in public (AOR = 2.73, 95%CI: 2.27–3.28) and private (AOR = 2.21, 95%CI: 1.63–2.98) health facilities, women living in the Southern East (AOR = 2.54, 95%CI: 1.84–3.52) and Central high (AOR = 1.71, 95%CI: 1.14–2.57) regions, those with ≥ 4 (AOR = 1.61, 95%CI: 1.28–2.02) and 1–3 (AOR = 1.26, 95%CI: 1.03–1.54) antenatal care (ANC) visits, and women belonging to households where the head had secondary/higher education (AOR = 1.23, 95%CI: 1.01–1.49).

**Conclusion:**

PNC utilization in rural Afghanistan is unacceptably low. It was significantly associated with place of delivery, region of residence, prior ANC attendance, and the household head's education. These findings highlight the importance of strengthening maternal health services by promoting ANC attendance, encouraging facility‐based deliveries, and addressing regional disparities.

**Abbreviations**
ANC: antenatal care
AOR: adjusted odds ratio
CI: confidence interval
COR: Crude odds ratio
LMICs: Low and middle‐income countries
MICS: Multiple Indicator Cluster Survey
MMR: maternal mortality ratio
PNC: postnatal care
PSU: primary sampling units
VIF: variance inflation factors
WHO: World Health Organization

## 1 Introduction

The postnatal period refers to the time from childbirth until 42 days postpartum, during which a significant proportion of maternal and newborn deaths occur. Immediately after delivery, the major threats to a mother's life are postpartum hemorrhage and infections, while newborns face the highest risks from preterm birth complications, birth asphyxia, and severe infections [1, 2]. Many of these complications can be prevented or effectively managed through timely and adequate utilization of postnatal care (PNC) services [3, 4]. Despite global efforts to improve maternal and neonatal health, the rates of maternal and neonatal mortality and morbidity during the postnatal period remain alarmingly high, particularly in low and middle‐income countries (LMICs) [4, 5].

Previous studies indicate that the utilization of PNC services is influenced by a variety of sociodemographic, economic, and health service‐related factors. These factors include women's educational status, maternal age, occupation, place of delivery, awareness of PNC services, attendance at antenatal care (ANC) visits, place of residence, parity, household wealth index, birth order, presence of skilled birth attendants during delivery, exposure to mass media, and mode of delivery (such as cesarean section) [6, 7, 8, 9, 10, 11, 12, 13, 14, 15]. In addition, a study conducted in rural areas of northwest Ethiopia reported that limited access to transportation was significantly associated with lower utilization of PNC services [16].

Afghanistan still has a maternal mortality ratio of approximately 638 deaths per 100,000 live births, one of the highest in the region [17]. Despite some improvements in recent decades, maternal and neonatal health indicators in the country remain among the most challenging in the region. Studies conducted in Afghanistan indicate that only 16.0% of women receive early PNC services [18]. Furthermore, women living in the southern and southeastern regions of the country are less likely to utilize PNC services compared with those living in other parts of the country [19]. However, there is limited evidence specifically examining the utilization of PNC services among women living in rural areas of Afghanistan.

Women residing in rural settings often face multiple barriers to accessing maternal health services, including limited availability of health facilities, shortage of skilled healthcare providers [20], low levels of education and awareness [21, 22], heavy physical workload, limited decision‐making power, short birth intervals, and security challenges [23]. In addition, the recent withdrawal of international funding and humanitarian support has further weakened the health system, potentially affecting access to essential maternal health services [24, 25]. As a result, women in rural areas are more likely to give birth at home and less likely to receive timely PNC services [26]. Additionally, in 2023, approximately one quarter of the Afghan population resided in underserved regions, primarily located in rural and mountainous areas [27].

Although Afghanistan has achieved notable reductions in maternal and overall mortality over the past two decades, significant disparities remain between urban and rural populations [28]. Rural women continue to experience limited access to essential maternal health services. Previous studies on maternal health in Afghanistan have primarily focused on ANC utilization [29, 30, 31] and institutional delivery [32, 33], while the utilization of PNC services, particularly in rural areas, has received relatively little attention. Despite the critical role of PNC services in preventing maternal and neonatal complications and deaths, there is still limited research examining the factors influencing the utilization of PNC services in rural Afghanistan. Understanding the determinants of PNC utilization is therefore essential for designing targeted interventions and improving maternal and newborn health outcomes in these underserved communities. Therefore, this study aimed to assess the utilization of PNC services and identify its associated factors among women in rural Afghanistan.

## 2 Methods

### 2.1 Study Design and Data Source

**Figure 1:**
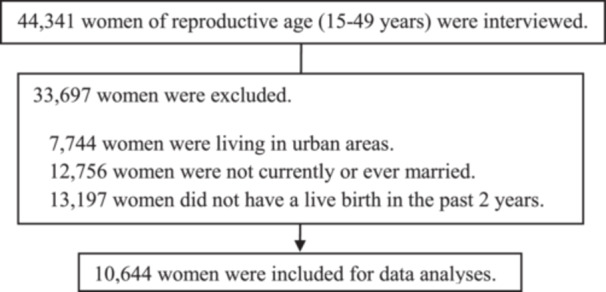
Final sample size and schematic presentation of the sample selection.

In this cross‐sectional study, we used data from the Afghanistan Multiple Indicator Cluster Survey (MICS) 2022–2023. The MICS 2022–2023 employed a two‐stage stratified cluster sampling design to collect data from households across Afghanistan. Sampling methodology and data collection approach are reported elsewhere [34]. As part of this national survey, data were collected from women of reproductive age (15–49 years) by trained surveyors [34]. For this analysis, we included ever‐married women aged 15–49 years who delivered a live child in the last 2 years prior to the survey and were residing in rural areas of Afghanistan. The final analytical sample consisted of 10,644 women (Figure 1).

### 2.2 Study Variables

#### 2.2.1 Outcome Variable

The outcome variable was PNC utilization and was defined as whether a woman received at least one health check after delivery following her most recent childbirth. The variable was constructed using responses to the survey questions “Was your health checked after childbirth?” (yes vs. no), and “How many times was your health checked after childbirth?” (once vs. more than once). Based on these responses, a binary outcome variable was created, indicating whether the woman received at least one PNC visit (yes vs. no).

#### 2.2.2 Independent Variables

The independent variables included woman age at the time of survey (15–24, 25–39, 40–49 years), woman education level (no formal education, primary education, secondary/higher education), head of household education level (no formal education, primary education, secondary/higher education), birth order (1st child, 2nd or higher), place of delivery (home, public health facilities, private health facilities), ANC utilization (no visit, 1–3 visits, and ≥ 4 visits), woman had media exposure (yes vs. no), wealth status (lowest quintile up to highest quintile), and regions of Afghanistan (Central, Central High, East, Northern East, Southern East, North, South, West), and media exposure. Media exposure was defined as “yes” if the woman watched TV daily, or the woman listened to the radio daily, or the woman read a newspaper, and as “no” if otherwise. Details on the construction of the variable on wealth status are provided in the MICS 2022–23 report [34].

#### 2.2.3 Statistical Analysis

For this analysis, we restricted the dataset to women residing in rural areas by using the residence variable (rural vs. urban) and excluded women living in urban areas (Figure 1). Descriptive statistics were used to examine the distribution of baseline characteristics of women. A chi‐square test was used to assess differences between characteristics of women who received PNC service and those who did not. For model selection, we selected independent variables after reviewing the literature. We specified and fitted a binary logistic regression model (see below).

$$\log\left(\frac{p_{i}}{1-p_{i}}\right)=\beta_{0}+\sum_{k=1}^{K}\beta_{k}X_{\mathrm{ik}}$$

Here, $p_{i}$ represents the probability that woman ireceived at least one postnatal health check, $X_{\mathrm{ik}}$ represents the explanatory variables, $\beta_{0}$ is the intercept, and $\beta_{k}$ represents the regression coefficient for explanatory variable k. Exponentiating a regression coefficient, $\text{exp}(\beta_{k})$, yields the corresponding odds ratio.

We ran the model for bivariate and multivariable analyses. We examined the likelihood of at least one PNC check after childbirth and reported crude odds ratios (CORs) and adjusted odds ratios (AORs) with 95% confidence intervals (95% CIs). Multicollinearity among independent variables was assessed using the variance inflation factor (VIF), and no evidence of problematic multicollinearity was observed. To account for the complex survey design of the MICS dataset, sampling weights, strata, and primary sampling units (PSUs) were applied in all analyses. All statistical analyses were conducted using Stata version 18 (StataCorp, College Station, TX, USA) [35]. A two‐sided *p*‐value of < 0.05 was considered statistically significant.

#### 2.2.4 Ethics Approval

The Research and Ethics Committee at the Department of Public Health, Faculty of Medicine, Kandahar University waived the ethical approval because secondary data from the MICS 2022–2023 were used and analyzed in this study. The original MICS survey was ethically approved by the Afghanistan Ministry of Public Health (MoPH). For the MICS 2022–2023, informed consent to proceed with the interviews was obtained from all participants. For children, informed consent was taken from their legal guardians. Moreover, all methods were carried out in accordance with the Declaration of Helsinki.

## 3 Results

**Figure 2:**
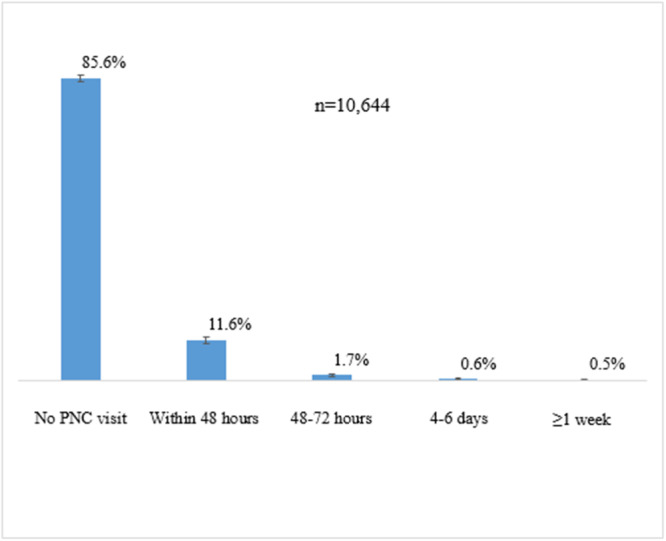
Weighted distribution of the timing of the first maternal postnatal health check after childbirth; error bars represent 95% confidence intervals.

Figure 2 shows that among the 10,644 women included in the study, 14.4% (95% CI 13.3%–15.4%) received at least one PNC service after childbirth, whereas 85.6% (95%CI 84.6–86.6%) did not receive any PNC service. The supplementary table presents the prevalence of PNC use at the national level, by region, and by province within each respective region in rural Afghanistan (See Supporting File 1).

The baseline characteristics of women according to PNC utilization status are presented in Table 1. Over 61.7% of women were 25–39 years old. Significant differences were observed between women who utilized PNC services and those who did not with respect to women's education level, education level of the household head, household wealth status, birth order, antenatal care (ANC) utilization, place of delivery, region of residence, and media exposure. For example, a higher proportion of women with secondary or higher education utilized PNC services compared with those who did not utilize PNC services (12.6% vs. 8.5%) (Table 1).

**Table 1:** Baseline characteristics of ever‐married women by status of postnatal care (PNC) utilization in rural Afghanistan.

Characteristics	Total *n* = 10,644 (%)	Postnatal care (PNC) used		*p*‐value
		No *n* = 8965 (%)	Yes *n* = 1679 (%)	
Women's age (years)				
15–24	30.6	30.4	31.8	0.66
25–39	61.7	61.8	60.7	
40–49	7.7	7.8	7.6	
Women's education				
No formal education	82.1	82.6	79.0	< 0.001
Primary	8.9	9.0	8.4	
Secondary/higher	9.1	8.5	12.6	
Household head education				
No formal education	67.9	69.0	61.7	< 0.001
Primary	11.9	12.1	11.1	
Secondary/higher	20.1	19.0	27.2	
Place of delivery				
Home	40.5	44.1	18.8	< 0.001
Public health facilities	51.4	48.0	71.9	
Private health facilities	8.2	8.0	9.3	
Antenatal care (ANC) visits				
No visit	25.6	27.3	15.4	< 0.001
1–3 visits	44.3	44.3	44.3	
≥ 4 visits	30.1	28.4	40.3	
Birth order				
1st child	15.3	14.7	19.0	< 0.001
2nd or higher child	84.7	85.3	81.0	
Access to media				
No	86.6	87.7	80.2	< 0.001
Yes	13.4	12.4	19.8	
Region of residence				
Central	10.5	10.2	12.3	< 0.001
Central high	3.7	3.3	5.8	
Eastern	12.6	12.3	14.2	
Southern East	11.6	9.5	23.9	
Northern East	13.6	14.1	10.5	
Northern	16.2	16.9	11.7	
Southern	17.7	18.5	12.3	
Western	14.3	15.1	9.3	
Wealth status				
Lowest quintile	20.1	21.2	13.8	< 0.001
Second	20.2	20.7	17.6	
Third	20.4	20.6	19.4	
Fourth	20.0	20.1	19.8	
Highest quintile	19.3	17.6	29.3	

Public health facilities refer to public hospitals, clinics, or health posts; and private health facilities refer to private hospitals or clinics. Percentages, confidence intervals, *p* values, and regression estimates account for the complex survey design and sampling weights.

The multivariable logistic regression results presented in Table 2 indicate several factors significantly associated with PNC service utilization. Place of delivery was significantly associated with PNC utilization. Women who delivered in public health facilities (AOR = 2.73, 95% CI: 2.27–3.28) and those who delivered in private health facilities (AOR = 2.21, 95% CI: 1.63–2.98) had significantly higher odds of utilizing PNC services compared with women who delivered at home. Regional differences in PNC utilization were also observed. Women residing in the Southern East (AOR = 2.54, 95% CI: 1.84–3.52) and Central High (AOR = 1.71, 95% CI: 1.14–2.57) regions had higher odds of utilizing PNC services compared with women residing in the Central region, which includes Kabul, Logar, Maidan Wardak, Parwan, Kapisa, and Panjshir provinces. Similarly, women who attended four or more ANC visits (AOR = 1.61, 95% CI: 1.28–2.02), women who attended 1–3 ANC visits (AOR = 1.26, 95% CI: 1.03–1.54) were more likely to utilize PNC services compared with women who did not attend any ANC visits. Women from households where the head of household had secondary or higher education were more likely to utilize PNC services compared with those whose household head had no formal education (AOR = 1.23, 95% CI: 1.01–1.49).

**Table 2:** Likelihood of utilizing postnatal care (PNC) by ever‐married women in rural Afghanistan.

Characteristics	COR (95%CI)	*p*‐Value	AOR (95%CI)	*p*‐value
Women's age (years)				
15–24	Reference		Reference	
25–39	0.94 (0.82–1.08)	0.36	1.00 (0.85–1.18)	0.96
40–49	0.93 (0.71–1.21)	0.59	1.07 (0.80–1.43)	0.65
Women's education				
No formal education	Reference		Reference	
Primary	0.98 (0.75–1.28)	0.89	0.90 (0.67–1.22)	0.50
Secondary/higher	1.55 (1.25–1.93)	< 0.001	1.12 (0.87–1.44)	0.38
Household head education				
No formal education	Reference		Reference	
Primary	1.03 (0.80–1.32)	0.81	0.93 (0.71–1.21)	0.58
Secondary/higher	1.60 (1.36–1.89)	< 0.001	**1.23 (1.01–1.49)**	**0.04**
Place of delivery				
Home	Reference		Reference	
Public clinic/hospital	3.52 (2.91–4.26)	< 0.001	**2.73 (2.27–3.28)**	**< 0.001**
Private clinic/hospital	2.75 (2.03–3.72)	< 0.001	**2.21 (1.63–2.98)**	**< 0.001**
Antenatal care (ANC) visits				
No visit	Reference		Reference	
1–3 visits	1.77 (1.44–2.17)	< 0.001	**1.26 (1.03–1.54)**	**0.02**
≥ 4 visits	2.52 (2.03–3.12)	< 0.001	**1.61 (1.28–2.02)**	**< 0.001**
Birth order				
1st child	Reference		Reference	
2nd or higher child	0.73 (0.61–0.89)	0.001	0.84 (0.67–1.05)	0.13
Access to media				
No	Reference		Reference	
Yes	1.75 (1.38–2.22)	< 0.001	1.21 (0.97–1.50)	0.09
Region of residence				
Central	Reference		Reference	
Central high	1.45 (1.00–2.09)	0.050	**1.71 (1.14–2.57)**	**0.01**
Eastern	0.95 (0.68–1.34)	0.786	1.16 (0.84–1.62)	0.37
Southern East	2.08 (1.52–2.85)	< 0.001	**2.54 (1.84–3.52)**	**< 0.001**
Northern East	0.62 (0.43–0.88)	0.01	0.81 (0.57–1.14)	0.23
Northern	0.57 (0.40–0.81)	< 0.001	0.71 (0.50–1.02)	0.06
Southern	0.55 (0.40–0.75)	< 0.001	0.89 (0.65–1.23)	0.49
Western	0.51 (0.35–0.75)	< 0.001	0.89 (0.59–1.32)	0.55
Wealth status				
Lowest quintile	Reference		Reference	
Second	1.30 (1.00–1.70)	0.049	1.11 (0.84–1.46)	0.47
Third	1.45 (1.13–1.85)	0.004	1.00 (0.77–1.30)	0.99
Fourth	1.51 (1.19–1.92)	0.001	0.87 (0.69–1.10)	0.24
Highest quintile	2.55 (1.91–3.40)	< 0.001	1.17 (0.87–1.57)	0.30

*Note:* Significant values are in bold.

Abbreviations: AOR; Adjusted odds ratio; COR; Crude odds ratio.

## 4 Discussion

This is the first study to determine the magnitude and determinants of PNC services utilization among women in rural Afghanistan. The findings show that PNC utilization in rural Afghanistan remains extremely low, with only 14.4% of women receiving at least one PNC visit after childbirth. Furthermore, household head's education, ANC utilization, place of delivery, and region of residence were significant predictors of PNC utilization.

Our study revealed that only 14.4% of women in rural Afghanistan utilized PNC services, a figure significantly lower than the national averages and urban estimates [19, 36]. Moreover, this rate was also lower than those reported in previous studies conducted in rural settings of some LMICs, such as 25% in Myanmar [37], 23% in western Ethiopia [38], and 33% in sub‐Saharan Africa [39]. Several factors may contribute to this suboptimal utilization, including widespread illiteracy, poverty, limited access to maternal healthcare services, and cultural beliefs and practices [32, 36, 40]. A recent qualitative study revealed that traditional practices, such as the seclusion of women following childbirth, remain prevalent in certain rural regions of Afghanistan [26]. Additionally, earlier research has also reported poor utilization of maternal healthcare services in rural Afghanistan [32, 33]. Moreover, statistics indicate that maternal morbidity and mortality are concentrated in rural regions of Afghanistan [25, 41, 42]. Given the well‐established benefits of PNC services in reducing maternal morbidity and mortality, there is an urgent need to design and implement targeted programs that can reduce barriers and improve access to and utilization of PNC services in rural Afghanistan.

To our knowledge, no newer nationally representative household survey has reported a directly comparable estimate of maternal PNC utilization after the 2022–2023 MICS. Nevertheless, more recent programmatic evidence suggests a mixed and fragile trajectory in maternal health service delivery. In 2025, UNICEF‐supported primary healthcare services reached more than 20 million people through 2409 health facilities and community platforms, enabled 1.5 million pregnant women to receive a first ANC visit and 905,801 births to be attended by skilled health personnel, and supported the development of national guidelines for ANC and PNC [43]. However, WHO reported that 167 health facilities had closed by March 2025 because of funding shortages, disrupting access to healthcare for approximately 1.6 million people, while more than 220 additional facilities were at risk of closure [44]. These developments indicate that efforts to strengthen maternal healthcare have continued, but service availability remains vulnerable, particularly in underserved areas. Updated population‐based data are therefore required to determine whether maternal PNC utilization has improved since the 2022–2023 survey.

The household head's educational level emerged as a factor influencing PNC service utilization in rural Afghanistan. Women in households with a head who had secondary or higher education were about 1.2 times more likely to use PNC services than those in households with a head with no formal education. This finding is consistent with previous studies conducted in LMICs that underscore the importance of household head education in shaping awareness regarding women's healthcare needs, including access and utilization of maternal healthcare services [37, 45, 46]. Earlier research in Afghanistan has also demonstrated that education of the household head can influence women's access to healthcare services by improving awareness of maternal health needs and increasing support for seeking care [30, 32, 33]. These findings highlight the broader influence of household socioeconomic and educational environments on maternal healthcare utilization in rural settings. In the current sociopolitical context of Afghanistan, community‐based health education programs targeting families and household decision‐makers may play an important role in improving awareness and demand for maternal health services, including PNC.

Consistentwith previous evidence, ANC utilization was positively associated with PNC utilization in this study. Women who attended 1–3 ANC visits or four or more ANC visits had significantly higher odds of utilizing PNC services compared with women who did not attend ANC visits. Similar associations have been reported in studies conducted across LMICs [8, 38, 39, 46]. ANC services often serve as an important entry point to the maternal healthcare continuum, where women receive health education, counseling, and referrals for subsequent services during childbirth and the postnatal period [21, 33, 36, 38, 46]. In Afghanistan, earlier studies have also documented a strong relationship between ANC attendance and subsequent use of maternal health services, including PNC [18, 19, 31, 32]. However, the overall quality and coverage of ANC services remain suboptimal in many rural areas of Afghanistan [29, 30, 33, 36, 47]. Strengthening ANC programs, particularly by improving service quality, health education, and continuity of care, may therefore contribute not only to improved pregnancy outcomes but also to increased utilization of PNC services.

Place of delivery also emerged as a significant predictor of PNC utilization. Women who delivered in public or private health facilities were significantly more likely to utilize PNC services compared with those who delivered at home. Similar findings have been reported in studies conducted in Ethiopia and other LMIC settings [8, 39, 48]. One possible explanation is that women who deliver in health facilities are more likely to receive immediate post‐delivery care, counseling on postpartum health, and information about follow‐up services, which may encourage subsequent use of PNC services. Previous studies in Afghanistan have also demonstrated that institutional delivery is strongly associated with the utilization of other maternal health services, including PNC [18, 19]. However, although institutional delivery has improved in recent years, a substantial proportion of women in rural Afghanistan still give birth at home, often due to geographic barriers, transportation challenges, and limited availability of skilled healthcare providers [32, 33]. Improving access to facility‐based delivery services, strengthening referral systems, and expanding community midwife programs may therefore play a key role in improving PNC utilization in rural areas.

Finally, the study identified substantial regional disparities in PNC utilization across rural Afghanistan. Women residing in the Central High and Southern East regions were more likely to utilize PNC services compared with women in the Central region. Regional differences in maternal healthcare utilization have also been reported in studies conducted in other LMICs [37, 39, 42]. In Afghanistan, previous research has similarly documented geographic inequalities in access to maternal and child health services, often driven by differences in health infrastructure, security conditions, availability of healthcare providers, and transportation networks [42, 47]. In addition, regional variation may reflect differences in social and cultural norms that shape women's autonomy to seek maternal health services. In some Afghan communities, women's ability to attend PNC services may depend on household decision‐making, permission to travel, availability of a male escort, and support from husbands or senior female relatives, including mothers‐in‐law and mothers [33, 49, 50]. Therefore, regional disparities in PNC utilization may partly reflect differences in women's decision‐making authority and freedom of movement, rather than service availability alone. However, because ethnicity, intra‐household decision‐making, and women's autonomy were not directly measured in this analysis, these explanations should be interpreted cautiously. In general, these findings suggest that maternal health interventions should be tailored to the specific needs and challenges of different regions. Further research, particularly mixed‐methods and qualitative studies, would be valuable for understanding the contextual barriers and facilitators influencing PNC utilization in specific rural regions of Afghanistan.

## 5 Limitations

This study provides important insights about PNC service utilization in rural Afghanistan. However, certain limitations deserve mentioning. First, the data collected for the MICS survey were self‐reported and, therefore, could be subject to information and recall biases. Second, additional predictors, such as distance to a healthcare facility, awareness of PNC visits, and history of adverse pregnancy outcomes, which were not collected in the MICS survey, could potentially influence the utilization of PNC services. Third, the study relied on a binary measure of PNC utilization defined as at least one postnatal check after childbirth, which does not capture the timing, frequency, or quality of PNC services received. Therefore, the measure used in this study may not fully reflect adherence to WHO‐recommended PNC contacts. Therefore, future studies should consider these metrics in their analyses. Finally, the cross‐sectional design of the study precludes causal inference regarding the relationship between the determinants and PNC service utilization.

Despite the limitations, this study has several strengths. It used nationally representative data from the Afghanistan MICS 2022–2023, allowing for robust estimates of PNC utilization among women living in rural Afghanistan. The large sample size and the use of survey‐weighted analyses that accounted for the complex sampling design enhance the validity and generalizability of the findings.

## 6 Conclusion

This study demonstrates that PNC service utilization among women living in rural Afghanistan remains critically low. The strongest adjusted associations were observed for place of delivery and region of residence, followed by ANC attendance and the education level of the household head. These findings underscore the importance of strengthening the continuum of maternal healthcare in rural Afghanistan by expanding access to quality ANC and facility‐based delivery services, improving maternal health awareness at the household level, and addressing regional inequalities in access to care. Targeted community‐based and health‐system interventions may contribute to improved maternal and newborn health outcomes in rural Afghanistan.

## Author Contributions

**Essa Tawfiq:** conceptualization, investigation, writing – original draft, writing – review and editing, formal analysis, data curation, software, methodology. **Muhammad Haroon Stanikzai:** conceptualization, investigation, writing – original draft, writing – review and editing; validation; methodology, software, formal analysis, project administration, data curation, supervision. **Zainab Ezadi:** investigation, conceptualization, writing – original draft, writing – review and editing; data curation, methodology. **Fateme Dadras:** writing – original draft, conceptualization, writing – review and editing, data curation, software. **Hadia Sayam:** conceptualization, investigation, writing – original draft, writing – review and editing, methodology, data curation. **Omid Dadras:** conceptualization, writing – original draft, investigation, writing – review and editing, validation, methodology, supervision, data curation, project administration, software.

## Funding

The authors have nothing to report.

## Conflicts of Interest

The authors declare no conflicts of interest.

## Author Declaration

All authors have read and approved the final version of the manuscript. Muhammad Haroon Stanikzai had full access to all of the data in this study and takes complete responsibility for the integrity of the data and the accuracy of the data analysis.

## Transparency Statement

Muhammad Haroon Stanikzai affirms that this manuscript is an honest, accurate, and transparent account of the study being reported; that no important aspects of the study have been omitted; and that any discrepancies from the study as planned (and, if relevant, registered) have been explained.

## Supporting information


Supporting File

## Data Availability

The MICS 2022–23 dataset is publicly available on UNICEF's official website through the following link: https://mics.unicef.org/surveys?display=card&keys=Afghanistan.
