# Supplementary material for: Postnatal Care Service Utilization and Associated Factors Among Women in Rural Afghanistan: A Cross‐Sectional Analysis of the 2022–2023 Multiple Indicator Cluster Survey
Source: Health Sci Rep. 2026 Jul 27;9(8):e72913. doi: 10.1002/hsr2.72913 (PMC13403044; doi:10.1002/hsr2.72913)
Supplement: Supplementary file 1 — Supporting File [file HSR2-9-e72913-s001.pdf]

Supplementary File 1. PNC utilization in rural areas by regions and provinces

N=10,644	Provinces	Prevalence (95% CI) %
National level		14.4 (13.3-15.4)
Central region		16.8 (13.3-20.4)
	Kabul	25.7 (13.6-37.8)
	Kapisa	8.0 (4.1-11.8)
	Parwan	30.7 (23.7-37.7)
	Maidan/Wardak	3.9 (1.5-6.2)
	Logar	5.2 (2.6-7.8)
	Panjshir	20.4 (14.5-26.3)
Central high region		22.7 (17.9-27.4)
	Bamyan	7.6 (2.8-12.4)
	Daykundi	27.5 (22.1-33.0)
Eastern region		16.2 (13.0-19.3)
	Nengarhar	13.7 (8.8-18.6)
	Laghman	13.2 (7.6-18.7)
	Kunarha	37.0 (30.4-43.6)
	Nooristan	2.4 (0.6-4.2)
Southern East region		29.6 (25.6-33.6)
	Ghazni	20.4 (14.4-26.4)
	Paktika	26.2 (21.9-30.5)
	Paktya	7.3 (4.1-10.5)
	Khost	58.1 (49.1-67.1)
Northern East region		11.1 (8.7-13.5)
	Badakhshan	14.7 (9.1-20.2)
	Takhar	5.4 (2.4-8.5)
	Kunduz	8.0 (3.7-12.2)
	Baghlan	19.9 (13.8-26.1)
Northern region		10.4 (8.1-12.6)
	Samangan	11.6 (6.3-16.9)
	Balkh	8.4 (3.3-13.4)
	Sar-e-pul	15.1 (8.8-21.4)
	Jawzjan	21.4 (17.3-25.6)
	Faryab	4.4 (1.7-7.2)
Southern region		10.0 (8.4-11.7)
	Uruzgan	4.4 (1.3-7.5)
	Zabul	2.6 (0.7-4.6)
	Kandahar	16.0 (12.5-19.4)
	Helmand	5.7 (3.6-7.8)
	Nimroz	40.5 (31.3-49.6)
Western region		9.4 (6.9-11.8)
	Ghor	5.7 (2.4-9.0)
	Badghis	24.9 (17.4-32.3)
	Herat	4.4 (0.9-7.9)
	Farah	11.0 (6.9-15.0)
